# Characterize neuronal responses to natural movies in the mouse superior colliculus

**DOI:** 10.3389/fncel.2025.1558504

**Published:** 2025-03-11

**Authors:** Ya-tang Li

**Affiliations:** Chinese Institute for Brain Research, Beijing, China

**Keywords:** superior collicullus, two-photon calcium imaging, natural movies, visual processing, neural encoding, unsupervised clustering

## Abstract

While artificial stimuli have been widely used in visual neuroscience and have significantly advanced our understanding of visual processing, they differ dramatically from the natural scenes that animals encounter in the wild. How natural stimuli are encoded in the superior colliculus (SC) and how neuronal responses to artificial and natural stimuli are related remain poorly understood. Here I applied two-photon calcium imaging to record neuronal activity in the mouse superficial SC in response to natural movies. An unsupervised learning algorithm grouped recorded neurons into 16 clusters based on their response patterns. Each cluster exhibited distinct temporal profiles, which arose from differences in both receptive field coverage and how neurons encode local visual features. Interestingly, I found a strong correlation between neuronal responses to natural movies and functional properties previously characterized using artificial stimuli. This suggests that the SC maintains a stable neural representation of visual information that is largely independent of the types of visual stimuli. Furthermore, neuronal responses to natural movies varied with depth within the superficial SC and across genetically defined neuronal types. These findings bridge the gap between our understanding of responses to artificial and natural stimuli, providing new insights into visual processing in the SC.

## 1 Introduction

Characterizing neuronal responses to diverse visual stimuli is a classical approach to understanding visual information processing in the brain. Theoretically, a neuron's functional properties could be fully described by analyzing its responses to every possible visual stimulus. However, the sheer number of potential stimuli renders this approach infeasible. Even a 10×10 pixel screen with 10 grayscale intensity levels per pixel yields 10^100^ possible combinations, a number 10^20^ times greater than the estimated number of atoms in the universe (10^80^). For a standard 600×800 monitor displaying 256 RGB colors, the number of combinations explodes to 256^3*600*800^ (~6*10^3, 460, 000^). Note that this calculation excludes temporal variations; considering continuous streams of static images—i.e. video, as experienced in daily life—further increases the number of possibilities. Practically, laboratory research typically employs reductionist approaches using specifically designed artificial stimuli, such as flashing spots for the retina (Kuffler, 1953) and moving bars for the visual cortex (Hubel and Wiesel, 1962).

Examining how neurons respond to artificial stimuli has proven to be a powerful and fruitful approach in neuroscience, significantly advancing our understanding of visual information processing. This approach has provided insights into both the neuronal encoding of visual information and the underlying neural circuits that implement these coding strategies. By isolating specific visual features, such as orientation or motion direction, researchers can effectively dissect the tuning properties of individual neurons. The efficacy of this approach is illustrated in a recent study on retinal ganglion cells (RGCs) (Baden et al., 2016). In this study, the authors employed an unsupervised clustering algorithm to analyze RGC responses to just four distinct visual stimuli. Surprisingly, they not only confirmed the existence of previously identified RGC types based on anatomical and molecular criteria, thus validating the approach, but also discovered new RGC types with distinct functional properties. Inspired by the success of this approach, we extended it to investigate the functional organization of the superior colliculus (SC), a midbrain structure evolutionarily conserved across all vertebrates (May, 2006). The SC's superficial layer receives direct inputs from RGCs and the primary visual cortex (V1), while its deeper layers play a crucial role in sensory integration and motor execution (Cang et al., 2018). We identified 26 distinct functional types (Li and Meister, 2023), a number comparable to the 43 cell types identified by gene expression profiling (Liu et al., 2023).

While artificial stimuli have proven invaluable, they differ substantially from the complex and dynamic natural scenes encountered by animals in their natural environments. Notably, the spectral power of natural images typically follows a power law relationship with spatial frequency (f), approximating 1/*f*^2^. From an evolutionary perspective, the ultimate purpose of animal visual systems is to enhance their survival and reproductive success in natural environments. Therefore, understanding how neurons process natural stimuli is important for a comprehensive understanding of visual function. A substantial body of research has recently emerged investigating neuronal responses to natural stimuli. For instance, researchers have employed two-photon calcium imaging to record neuronal responses to a large set of natural images from the ImageNet database (Deng et al., 2009) in visual cortex (Wang et al., 2024). This provides insights into how cortical neurons represent complex natural scenes. Electrophysiological recordings of neuronal responses to natural movies have also been performed in the primate SC (White et al., 2017; Bogadhi and Hafed, 2023), offering a further understanding of how dynamic visual information is processed. Despite these advancements, our understanding of neuronal responses to natural scenes, particularly natural movies, in mouse SC remains limited. Importantly, the relationship between neuronal responses to natural movies and the well-established functional properties derived from responses to artificial stimuli is still poorly understood.

To address this gap, I recorded responses to four distinct natural movies and a battery of artificial stimuli from the same population of SC neurons. This approach enables a direct comparison of responses to both stimulus types at the level of individual cells, bridging the gap between these two important lines of investigation. While our previous work revealed functional properties based on artificial stimuli, the present study focuses on responses to natural movies and their relationship to these established functional properties.

I identified 16 distinct clusters of neuronal responses to natural movies, each characterized by unique temporal profiles. These profiles captured the rich diversity of evoked neuronal dynamics. Neurons within these clusters exhibited distinct receptive field (RF) properties, including differences in the proportion of neurons with clearly defined RFs and variations in the spatial extent of RF coverage. The analysis revealed that RF properties partially accounted for the clustering of neurons based on their temporal responses to natural movies, indicating a link between spatial tuning and temporal dynamics. Importantly, neuronal responses to natural movies were strongly correlated with functional properties based on artificial stimuli. This robust relationship provides compelling evidence for a stable neural representation of visual information in the SC. Lastly, I observed distinct response patterns to natural movies across depth within the superficial SC and across genetically defined neuronal types.

## 2 Results

### 2.1 Diverse neuronal responses to natural movies in awake SC as revealed by two-photon calcium imaging

**Figure 1:**
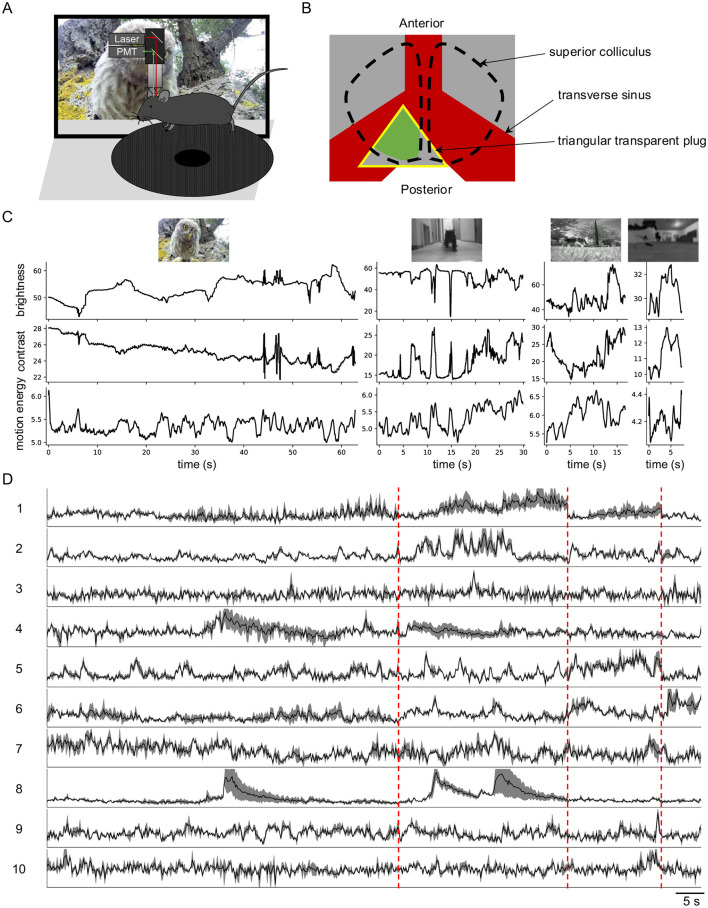
Diverse neuronal responses to natural movies in awake mouse superior colliculus. **(A)** Schematic of the experimental setup. Mice were head-fixed and free to run on a circular treadmill. Natural movies were presented on a screen. Neuronal calcium activity was imaged using two-photon microscopy. PMT, photomultiplier tube. **(B)** Schematic of mouse brain anatomy after insertion of a triangular transparent plug to expose the posterior-medial SC underneath the transverse sinus. The yellow triangle indicates the plug. The green color indicates the expression of GCaMP6. **(C)** The time-varying brightness, contrast, and motion energy of each natural movie. **(D)** Response profiles of 10 example neurons to four natural movies. Each row is scaled to the maximal response. Gray shading indicates the standard deviation. Vertical red dashed lines separate responses to different movies.

To investigate how SC neurons encode information in natural visual inputs, I used two-photon microscopy to image neuronal calcium responses to natural movies (Figure 1A). To maintain an intact cortex, the posterior-medial SC was exposed by gently pushing forward the transverse sinus (Li et al., 2023) (Figure 1B). In total, I imaged 3,414 neurons with reliable responses (SNR > 0.35) from 41 image planes in 16 animals. These animals were from six different mouse lines: wild-type, Vglut2-Cre (excitatory neurons), Vgat-Cre (inhibitory neurons), Tac1-Cre, Rorb-Cre, and Ntsr1-Cre (wide-field cells).

Neurons in SC exhibited robust and diverse responses to four distinct natural movies (Figures 1C, D). This response diversity was not attributable to animal locomotion, as the animals remained largely stationary during visual stimulation, and locomotion has minimal effects on SC neuronal responses (Savier et al., 2019).

### 2.2 Clustering neuronal responses to natural movies into 16 groups

**Figure 2:**
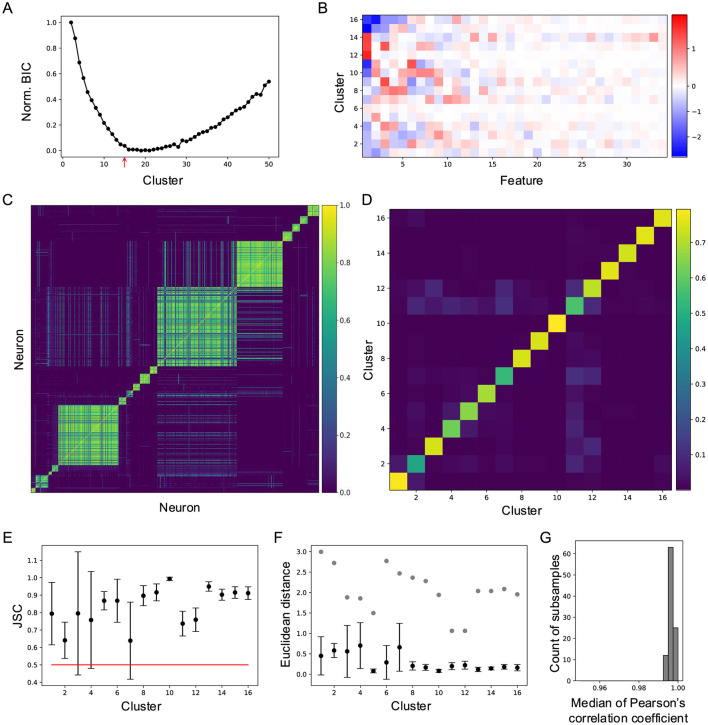
Cluster neuronal responses to natural movies into 16 groups. **(A)** Normalized Bayesian information criterion for Gaussian mixture models with different numbers of clusters. **(B)** Feature-weights for the 16 clusters. The color bar indicates the weights of features for each cluster. **(C)** Cell-wise co-association matrix (see Section 4). The color bar indicates the co-clustering fraction. **(D)** Between-cluster rate, which is the cluster-wise average of the co-association matrix. **(E)** Jaccard similarity coefficient (JSC) between the full dataset and subsets (Mean ± SD). **(F)** Euclidean distance between the original clusters and clusters identified on the subsets (Mean ± SD), black symbols. Gray symbols indicate the shortest Euclidean distance between the original cluster and other clusters. **(G)** Histogram of median correlation coefficients between the original clusters and clusters identified on 100 subsets.

To quantify neuronal responses to natural movies, I applied principal component analysis (PCA) to the raw response traces, reducing the data to a 3,414 × 34 matrix in the feature space. This feature matrix was then fitted with a Gaussian mixture model (GMM) using varying numbers of clusters (Figure 2). The quality of each clustering was assessed with the Bayesian information criterion (BIC; Figure 2A). The BIC decreased monotonically until the number of clusters reached 16, after which it increased slowly. This indicates that a 16-component GMM provides the best fit for the neuronal responses in feature space (Figure 2B), suggesting that neuronal responses to natural movies are best characterized by 16 clusters.

Next, cluster stability was assessed using sub-sampling analysis. I randomly sampled 90% of the data 100 times and fitted a GMM with 16 components to each sub-sample. For every neuron pair, I counted how often they were assigned to the same cluster across these 100 sub-samples (Figure 2C). The average co-clustering frequency for most clusters exceeded 0.5 (Figure 2D), indicating consistent clustering across subsets. To further quantify this stability, I calculated the Jaccard similarity coefficient (JSC), defined as the ratio of the intersection to the union of the cluster assignments. This analysis revealed high stability for all clusters (Figure 2E).

Additional evidence of stability was provided by comparing Euclidean distances between clusters. The distances between corresponding clusters in the subsets and the original dataset were smaller than the distances between distinct clusters within the original dataset (Figure 2F). Stability was further validated by the correlation between original clusters and clusters identified in the subsets (Figure 2G). Collectively, these results demonstrate that the observed cluster stability surpasses that reported in related studies (Baden et al., 2016; Li and Meister, 2023).

### 2.3 Characterize neuronal responses to natural movies across the 16 clusters

**Figure 3:**
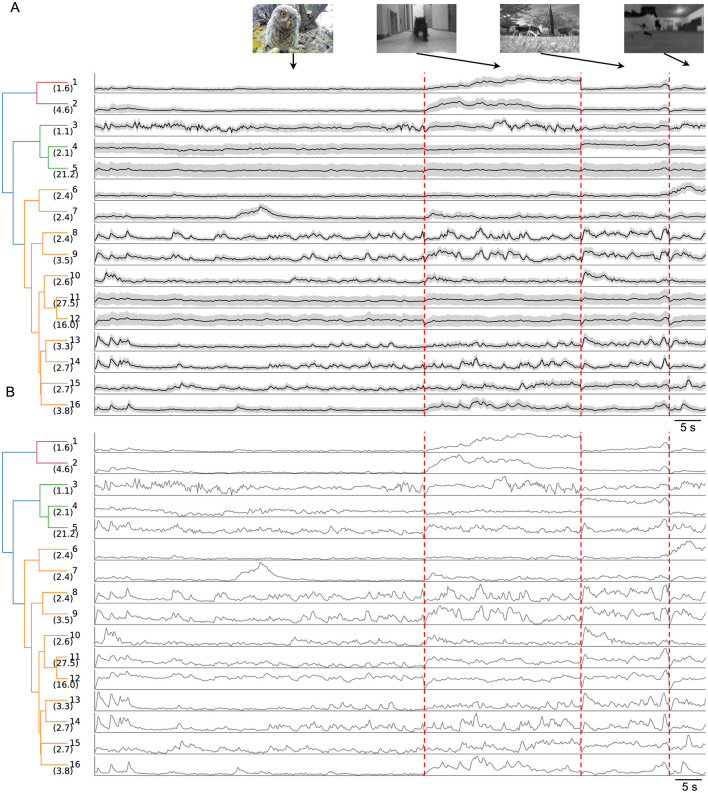
Temporal profiles of neuronal responses across the 16 clusters. **(A)** Dendrogram of 16 clusters based on their distance in feature space, with each row showing the average temporal profiles of neuronal responses to four natural movies. Gray shading indicates the standard deviation. The vertical scale is identical for all types and stimulus conditions. Vertical red dashed lines separate responses to different movies. **(B)** Normalized average temporal profiles.

To uncover the specific information about the natural movies conveyed by the 16 clusters, I analyzed the average temporal profiles of each cluster (Figure 3). A dendrogram, based on feature-space distances between cluster centers, illustrated the hierarchical relationships among these clusters.

Certain clusters exhibited strong preferences for specific movies. For example, Clusters 1 and 2 responded robustly to the “running cat” movie with distinct temporal profiles while showing weaker responses to other movies. Similarly, Cluster 4 preferred the “foraging” movie, Cluster 6 the “optical flow” movie, and Cluster 7 the “baby owl” movie. This movie selectivity indicates that SC neurons contribute to encoding visual categories. Such high-level processing by the SC is further supported by findings in primates, where SC neurons exhibit a preference for faces with short latency (Yu et al., 2024).

Most other clusters displayed robust yet transient responses to all natural movies, except for Cluster 12, which showed sustained activity throughout each movie. The transient responses likely reflect the RF properties of individual neurons, as each neuron's RF covers only a small portion of the visual stimulus.

**Figure 4:**
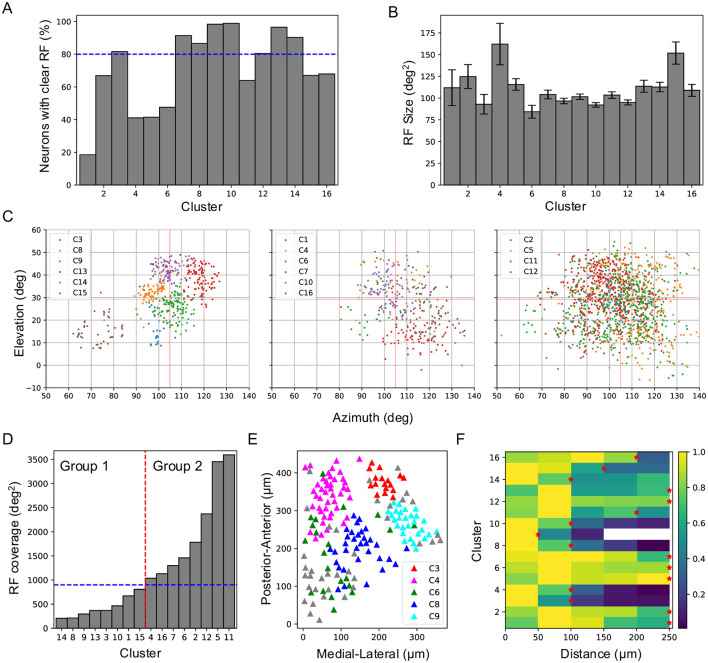
Receptive field properties and anatomical organization across clusters. **(A)** Percentage of neurons in each cluster that show clear RFs to flash stimuli. The blue dashed line marks 80%. **(B)** RF size for each cluster (mean ± SD). **(C)** RF positions of all clusters plotted in three panels based on their RF coverage. **(D)** Sorted RF coverage for all clusters. The vertical red dashed line separates clusters into two groups, and the blue dashed line marks 900 deg^2^. **(E)** Anatomical locations of different clusters of neurons in an example imaging field. **(F)** Neuron density within each functional cluster as a function of distance from a neuron of the same cluster. Red stars indicate the smallest radius at which the neuron density exceeds half of the peak density.

To determine how RF properties contribute to responses to natural movies, I mapped the RFs of individual neurons using flashing sparse noise. Only about 67% of SC neurons were found to have clearly defined RFs, and this percentage varied substantially across clusters, ranging from 19% in Cluster 1 to 99% in Cluster 10 (Figure 4A). RF size ranged from 84 deg^2^ to 162 deg^2^ (Figure 4B), and RF coverage also varied (Figures 4C, D). Based on RF coverage, the clusters were divided into two groups (Figure 4D). Group 1 comprised eight clusters with RF coverage below 900 deg^2^. These clusters occupied distinct parts of the visual field, suggesting their responses primarily reflected local features of the movies. Clusters with the smallest spatial coverage (e.g., Clusters 8, 9, 13, and 14) exhibited distinct temporal profiles (Figure 3B), largely because their RFs covered different regions of the natural movies. The difference between Clusters 3 and 15 was more pronounced than that between Clusters 13 and 14, as their RFs were spatially more distant. On the other hand, although the RFs of Cluster 10 overlapped with those of Clusters 8, 9, and 14, their temporal profiles were distinct, suggesting that they encoded different aspects of local features within a larger visual field. This role in local feature encoding is supported by the high proportion of neurons with well-defined RFs in these clusters (Figure 4A). Conversely, Cluster 1, with only 10 of 54 neurons having clear RFs, likely did not encode local features, and its small RF coverage may be attributed to this low sampling number.

Group 2 included clusters with larger and largely overlapping RF coverage. Clusters 2, 5, 11, and 12 exhibited the largest spatial coverage, with RFs spanning nearly the entire recorded visual field. Clusters 4, 6, and 16 also exhibited large spatial coverage and had few neurons with clear RFs, except for Cluster 7. Over 80% of the neurons in Cluster 7 showed clear RFs, and this cluster covered a visual region distinct from those covered by Group 1 clusters, suggesting a role in encoding local visual features.

The influence of RF properties on clustering was further supported by the anatomical organization of each cluster. Clusters with smaller RF coverage occupied more compact anatomical regions (Figure 4E). Density recovery profile analysis (Rodieck, 1991; Zhang et al., 2012) showed that neuron density decreased with distance for most clusters (Figure 4F), indicating that neurons within the same cluster form localized patches, similar to previously identified functional types (Li and Meister, 2023). For Group 1 clusters, neuron density dropped to half its peak value at a radius of ~ 100 μ m (~ 25° diameter in visual space), consistent with RF coverage and reinforcing the role of RF properties in the clustering process. In Group 2, neurons in most clusters also form patches, though with larger patch sizes, reflecting their broader RF coverage.

### 2.4 Functional properties of the 16 clusters of neurons

**Figure 5:**
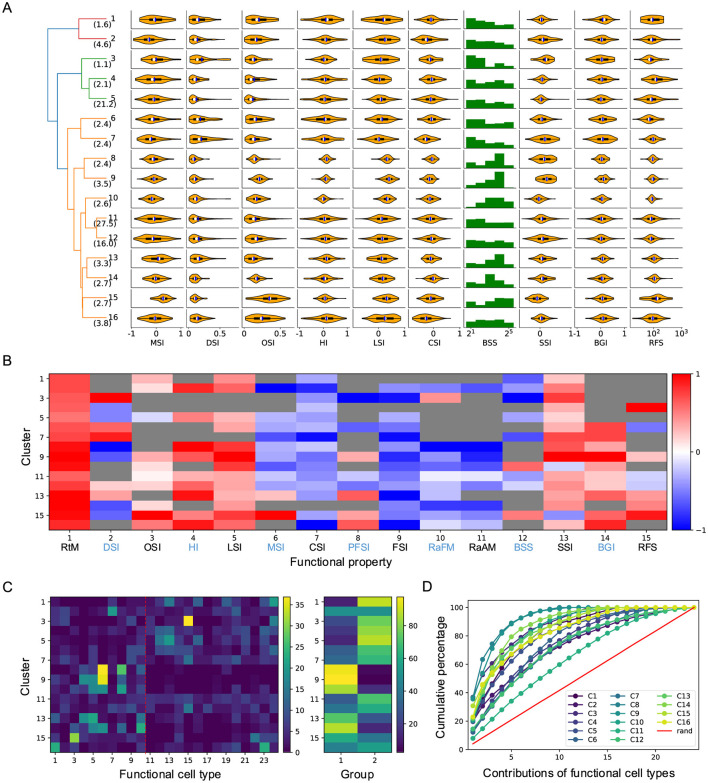
Functional properties of each cluster. **(A)** Violin plots or histograms of various response indices: motion selectivity index (MSI), direction selectivity index (DSI), orientation selectivity index (OSI), habituation index (HI), looming selectivity index (LSI), contrast selectivity index (CSI), best stimulus size (BSS), surround suppression index (SSI), blue-green index (BGI), and receptive field size (RFS). **(B)** Selectivity index (SI, normalized for each column) for functional properties across different clusters (see Section 4). RtM, response to motion; PFSI, peak-final selectivity index; FSI, frequency selectivity index; RaFM, response after frequency modulation; RaAM, response after amplitude modulation. The gray color indicates values that are not significantly different from 0 (*p* ≥ .05, one-sample *t*-test). **(C)** Contributions of different functional types to each cluster. The red dashed line divides cell types into two groups, as shown in the right panel. **(D)** Cumulative percentage of contributions of different functional types to each cluster.

In our previous work, we identified 24 functional cell types based on neuronal responses to artificial stimuli in the superficial SC (Li and Meister, 2023). We quantified their functional properties (see Materials and methods) and demonstrated functional diversity across these types. Thus, the clustering of neuronal responses to natural movies likely reflects not only differences in RF properties but also functional diversity. Supporting this idea, the results revealed diverse functional properties across clusters (Figures 5A, B). For example, although Clusters 8 and 9 spatially overlapped with Cluster 10, they exhibited distinct functional properties, including greater habituation to looming stimuli, sharper size tuning, and stronger surrounding suppression. Similarly, despite covering large and overlapping visual regions, Clusters 5 and 12 displayed different functional properties. Compared to Cluster 5, Cluster 12 showed stronger direction selectivity and smaller RF size. Functional differences were also observed among clusters with largely non-overlapping RF coverage. For example, Cluster 15, with its large RFs, exhibited higher orientation selectivity and a preference for large, moving stimuli, whereas neurons in Cluster 7, with smaller RFs, preferred small, flashing stimuli.

Next, I analyzed the relationship between these clusters and functional cell types. Surprisingly, despite the distinct nature of these two stimulus sets, a strong correlation emerged. The contribution of functional cell types to each cluster differed significantly from random expectations and varied dramatically across clusters (Figures 5C, D). In 14 of the 16 clusters, half of the functional types accounted for 80% of the contribution. Moreover, in 11 of the 16 clusters, five functional types accounted for over 50% of the contribution, with the largest contribution reaching 89%. Clusters with small RF coverage were predominantly composed of functional cell types from Group 1 (1–10), whereas other clusters were primarily associated with functional types from Group 2 (Figure 5C). These findings suggest a strong correlation between clusters defined by responses to natural movies and functional types identified through artificial stimuli. This relationship points to a stimulus-independent internal representation of visual information in the SC.

### 2.5 Natural movie elicited responses across depth and in genetically labeled neurons

**Figure 6:**
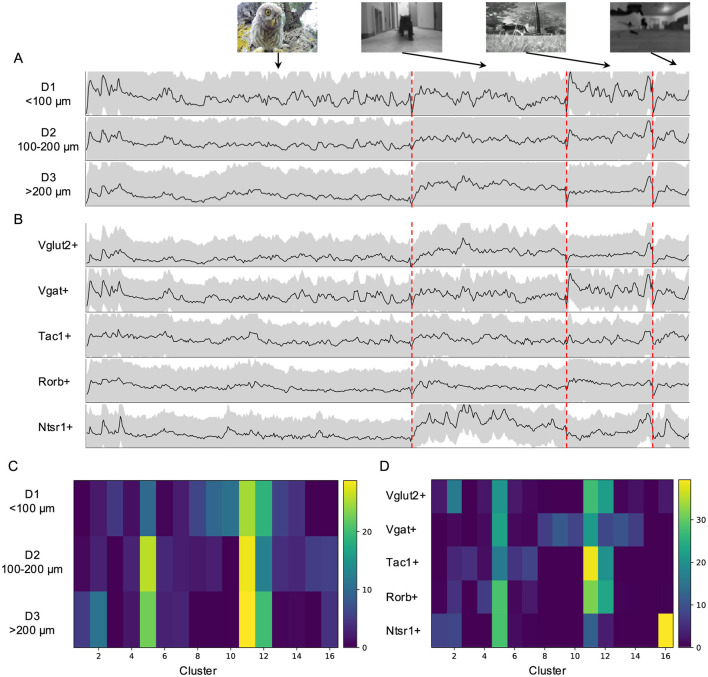
Natural movie elicited responses in genetically labeled populations and across depth. **(A)** Average temporal profiles of neuronal responses to natural movies of five genetically labeled cell types plotted as in Figure 3A. **(B)** Average temporal response across depth. **(C)** The percentage of each cluster across depth. **(D)** The percentage of each cluster in mice with different genetic backgrounds.

Our previous work has revealed depth-dependent variations in the functional properties of SC neurons (Li and Meister, 2023). Here, I explored whether such depth dependence also extends to neuronal responses to natural movies. Neurons in the upper 100 μm showed stronger responses to natural movies, particularly to the “baby owl” and “foraging” movies (Figure 6A), although their temporal profiles were largely consistent across depth. Compared with deeper neurons, superficial neurons were more strongly associated with clusters with smaller visual field coverage (Figure 6C).

I further compared responses to natural movies in genetically labeled neurons across five mouse Cre-lines (see Section 4). These neurons displayed distinct temporal profiles based on their genetic identity (Figure 6B). For example, excitatory (Vglut2+) and inhibitory (Vgat+) neurons exhibited different response patterns to the “baby owl” and “foraging” movies, with inhibitory neurons showing larger initial response amplitudes to both. Tac1+ and Rorb+ neurons displayed more sustained responses, whereas Ntsr1+ neurons, characterized by wide-field dendritic arbors, responded transiently to the “baby owl” movie and strongly to the “running cat” movie. This suggests that Ntsr1+ neurons may function as approach detectors.

The contribution of the 16 clusters to genetic lines also varied (Figure 6D). Ntsr1+ neurons were primarily associated with Clusters 5 and 16, which contained fewer neurons with clearly defined RFs and covered large visual regions. In contrast, Vgat+ neurons were more associated with clusters with small RF coverage. Meanwhile, Vglut2+, Tac1+, and Rorb+ neurons were contributed by clusters covering larger visual fields, though their specific contributions varied. Finally, I observed similarities in responses between Vgat+ neurons and neurons located in the upper 100 μm (D1), consistent with previous findings that Vgat+ neurons are more abundant in the upper portion of the superficial SC (Liu et al., 2023).

## 3 Discussion

In the wild, animals are exposed to natural scenes; however, in the laboratory, artificial stimuli are widely employed. Although such stimuli have provided insights into the neural processing of visual information, there is a gap between these laboratory findings and the processing of ecologically relevant natural scenes. This remians Bridging this gap is a central question in visual neuroscience. Our previous work quantified neuronal functional properties based on responses to artificial stimuli and revealed distinct functional cell types (Li and Meister, 2023). Building on this, the present study investigates how SC neurons respond to natural movies. By comparing responses to both natural and artificial stimuli within the same neuronal population, I aim to bridge the gap.

### 3.1 Main findings

I summarize the main findings as follows. Neurons in the superficial SC show diverse responses to four natural movies (Figure 1). Based on their temporal response profiles, these neurons are grouped into 16 clusters (Figure 2), each displaying distinct response patterns (Figure 3). The differences in temporal profiles are partially attributed to the RF properties of neurons in each cluster, and neurons in some clusters form clear patchy structures in both visual and anatomical spaces (Figure 4). Responses to natural movies are highly correlated with functional properties based on responses to artificial stimuli, indicating that the dimensionality of the neuronal response space is much lower compared to the stimulus space (Figure 5). Furthermore, the diversity in neuronal responses is observed across different depths and genetically defined cell types (Figure 6).

### 3.2 Interpretation of neuronal responses to natural movies

How do we interpret the neuronal responses to natural movies? The average RF size of SC neurons is about 100 deg^2^ (Figure 4B), covering around 1/30 of the visual area spanned by all recorded neurons (Figure 4C). In our previous study, artificial visual stimuli were centered at the RF center of each neuron, ensuring that differences in their responses reflected how they encode different visual features within the same input. In the present work, different neurons “see” distinct 1/30 portions of natural movies, meaning that differences in their responses reflect both encoding differences and distinct visual inputs. Indeed, neurons in some clusters occupied only small localized visual regions. However, a close look reveals that this explanation does not fully capture the observed response patterns.

First, several clusters respond preferentially to specific movies, including Clusters 1, 2, 4, 5, and 7. These neurons account for about 13% of the total recorded population and are mainly located in the deeper part of the superficial SC. This finding is consistent with the notion of a depth-dependent sifting of visual information within the SC (Lee et al., 2020).

Second, even for clusters with largely overlapping RF coverage (Figure 4C), they can respond very differently to the same stimulus. This reflects variations in their functional properties, such as orientation selectivity, motion direction selectivity, and size tuning. Therefore, a neuron's responses to natural movies can be largely explained by its RF location and functional properties.

### 3.3 Functional significance and the underlying neural mechanism

The diverse responses to natural movies observed in the superficial SC align with the parallel processing of visual information that begins in the retina, where more than 30 types of RGCs have been identified (Baden et al., 2016; Sanes and Masland, 2015). This diversity suggests the SC not only inherits but also transforms retinal inputs. For example, most clusters in Group 1 (Figures 4C, D) consist of neurons with small, well-defined RFs that cover localized visual regions, suggesting they encode low-level features inherited from the retina, such as orientation selectivity, direction selectivity, and surround suppression. In contrast, several clusters respond preferentially to specific movies, indicating a role in high-level visual processing, such as categorizing visual stimuli. Furthermore, neurons in Cluster 12 show persistent activity after switching to a new movie, suggesting their potential involvement in encoding brain states or working memory.

Additionally, the superficial SC is known to play a critical role in encoding stimulus-driven visual saliency, a key component of bottom-up attention (Barchini et al., 2018; Yan et al., 2018; White et al., 2017; Wu et al., 2024; Ahmadlou et al., 2017; Knierim and van Essen, 1992). Thus, neuronal responses to natural movies may not only reflect specific visual features or categories but also the saliency of visual locations. This saliency encoding is essential for guiding attention and gaze shifts, enabling animals to navigate and survive in complex natural environments.

The diverse responses to natural movies in the SC may arise from an integration of retinal, collicular, and cortical circuits, further influenced by neuromodulators. As noted, some response properties likely originate directly from the retina. However, retinal inputs are not passively relayed; they are integrated with collicular inputs from local excitatory and inhibitory neurons. Excitatory and inhibitory neurons show distinct temporal response profiles (Figure 6B) and contribute to different clusters (Figure 6D), suggesting they play complementary roles in shaping neuronal responses. The observation that some clusters contain both excitatory and inhibitory neurons may be attributed to two factors. First, because both types of neurons may receive direct retinal inputs or relayed inputs from local collicular neurons, the observation likely reflects their shared inputs. Second, the number and diversity of natural movies used in this study were limited. While retinal inputs dominate low-level feature encoding, cortical inputs likely drive higher-order functions. In the primate SC, face preference requires cortical inputs (Yu et al., 2024). Similarly, the category-specific responses observed here may also depend on inputs from V1. Furthermore, the persistent activity in Cluster 12 suggests contributions from neuromodulators like dopamine and serotonin (Bolton et al., 2015; Mooney et al., 1996), which regulate brain state and working memory.

### 3.4 From artificial to natural: bridging the gap in visual encoding

One way to probe the dimensionality of neural space in visual processing is to measure neuronal responses to a diverse set of visual stimuli. However, the number of possible visual stimuli is enormous, and even carefully selected natural movies with diverse content and style require a fairly long time to display. Moreover, the same natural movies must be displayed multiple times to elicit reliable neuronal responses. To further examine whether these responses are ethologically relevant, additional manipulations—such as rotating the movies or playing them in reverse—are necessary. These requirements collectively increase the recording time substantially. In the present study, imaging neuronal responses to the current set of stimuli took over two hours, during which the mice's eyes occasionally became blurred due to eye discharge. Prolonged imaging sessions would further increase this likelihood and lead to photo-bleaching of the fluorescence.

Therefore, the smaller number of clusters identified in this study, compared to the number of functional cell types reported before (Li and Meister, 2023), does not necessarily imply that natural movies are less effective in revealing the functional diversity of SC neurons, nor does it suggest that the neuronal representations of visual information are unstable. Instead, the discrepancy may simply reflect the limited number of natural movies used in the experiment.

A critical test for the relevance of artificial stimuli to natural visual processing is the ability to predict neuronal responses to natural movies based on their functional properties derived from responses to artificial stimuli. If such predictions are successful, it would strongly support the idea that what we have learned from artificial stimuli is valuable for understanding how the brain processes natural scenes. This would bridge the gap between controlled experimental paradigms and the complexity of real-world visual processing, offering a generalizable framework for studying neural representations of visual information.

## 4 Materials and methods

### 4.1 Animal

Six strains of laboratory mice of both sexes were used at ages 2–4 months: C57BL/6J (wild-type), Vglut2-ires-Cre (B6J.129S6(FVB)-*Slc17a6*^*tm2(cre)Lowl*^/MwarJ, JAX: 028863), Vgat-ires-Cre (B6J.129S6(FVB)-*Slc32a1*^*tm2(cre)Lowl*^/MwarJ, JAX: 028862), Tac1-IRES2-Cre-D (B6; 129S-*Tac1*^*tm1.1(cre)Hze*^/J, JAX: 021877) (Harris et al., 2014), Rorb-IRES2-Cre-D (B6;129S-*Rorb*^*tm1.1(cre)Hze*^/J, JAX: 023526) (Harris et al., 2014), and Ntsr1–GN209–Cre (Genset: 030780-UCD) (Gerfen et al., 2013). All animal procedures were performed according to the relevant guidelines and approved by the Caltech IACUC.

### 4.2 Viral injection

Adeno-associated virus (AAV) expressing non-floxed GCaMP6 (AAV2/1.hSyn1.GCaMP6f.WPRE.SV40, 10^12^ gc/ml) was injected into the SC of wild-type mice, while AAV expressing floxed GCaMP6 (AAV2/1.Syn.Flex.GCaMP6f.WPRE.SV40, 10^12^ gc/ml) was injected into the SC of Vglut2-ires-Cre, Vgat-ires-Cre, Tac1-IRES2-Cre-D, Rorb-IRES2-Cre-D, and Ntsr1–GN209–Cre mice. The injection site was 0.5 mm lateral and 0.42 mm anterior to the lambda, with injections performed at depths of 1 and 1.6 mm. After 2–3 weeks, a cranial window coupled to a transparent silicone plug was implanted to expose the posterior-medial portion of SC, which corresponds to the upper-temporal part of the visual field (Li et al., 2023). Two-photon microscopy was used to image calcium signals in the SC of head-fixed awake mice between 3 weeks and 2 months after viral injection.

### 4.3 *In vivo* two-photon calcium imaging

For imaging experiments, the mouse was head-fixed and free to move on a rotating treadmill. Two-photon imaging was performed on a custom-built microscope with a 16 × objective lens, 0.8 NA, 3 mm WD (Nikon). A Ti-Sapphire laser (Spectra-Physics Mai Tai HP DeepSee) was scanned by two galvanometers (Cambridge). GCaMP6f was excited at 920 nm, with laser power at the sample plane ranging from 20 to 80 mW. A 600 μm × 600 μm field of view was scanned at 4.8 Hz with a spatial resolution of 24 μm/pixel. The imaging depth was up to 350 μm. Emitted light was collected with a T600/200dcrb dichroic mirror (Chroma), passed through an HQ575/250m-2p bandpass filter (Chroma), and detected by a photomultiplier tube (R3896, Hamamatsu). Artifacts of the strobed stimulus (see below) were eliminated by discarding eight pixels on either end of each line. The animal's locomotion on the treadmill and pupil positions were recorded and synchronized to the image acquisition. The animal exhibited only rare eye movements and locomotion (Li et al., 2020).

### 4.4 Visual stimulation

An LED-backlit LCD screen was placed 18 cm away from the mouse's right eye. The center of the monitor was at 95° azimuth and 25° elevation to the eye, and the monitor covered a visual field of 106° × 79°. The monitor's LED illuminator was strobed for 12 μs at the end of each laser scan line to minimize interference from visual stimulation with fluorescence detection. The monitor was gamma-corrected. Four distinct natural movies, including “baby owl,” “running cat,” “foraging,” and “optical flow,” were presented to probe how collicular neurons responded to natural stimuli. The characteristics of natural movies were analyzed by calculating brightness, root mean square (RMS) contrast, and motion energy (Nishimoto et al., 2011).

Functional properties of the same neurons were characterized based on their neuronal response to six types of artificial visual stimuli (Li and Meister, 2023), including (1) A full-field moving black bar (5° width at 50°/s) in 12 directions to measure the orientation selectivity and direction selectivity. (2) An expanding black disc (diameter 2° to 60° at a speed of 60°/s, stationary at 60° for 0.25 s, followed by a gray background for 2 s) and a receding white disc (60° to 2° at a speed of 60°/s) to measure looming-related responses. (3) Sparse (one at a time) 5° × 5° flashing squares (11 × 11 squares, 1 s black or white + 1 s gray) to map the receptive field (RF); (4) A 10° × 10° square modulated by a “chirp” in frequency or amplitude [3 s black + 3 s white + 3 s black + 3 s gray + 8 s frequency modulation (2^−1:3^ Hz) + 3 s gray + 8 s amplitude modulation (0:1) + 3 s gray + 3 s black] centered on the RF to measure temporal properties (Baden et al., 2016); (5) A 10° × 10° square flashing blue or green (1 s black + 3 s blue + 4 s black + 3 s green + 3 s black) centered on the RF to measure the color preference; (6) A flashing disc (2 s black + 2 s gray) with different size (2°, 4°, 8°, 16°, 32°) centered on the RF to measure the size tuning. All stimuli were displayed for 10 repetitions.

### 4.5 Analysis of calcium responses

#### 4.5.1 Measurement of calcium responses

Brain motion during imaging was corrected using SIMA (Kaifosh et al., 2014) or NoRMCorre (Pnevmatikakis and Giovannucci, 2017). Regions of interest (ROIs) were drawn manually using the Cell Magic Wand Tool (ImageJ) and fitted with an ellipse in MATLAB. Fluorescence traces of each ROI were extracted after estimating and removing contamination from surrounding neuropil signals as described previously (Li et al., 2020; G'´obel and Helmchen, 2007; Kerlin et al., 2010). The true fluorescence signal of a neuron is *F*_true_ = *F*_raw_−(*r*·*F*_neuropil_), where *r* is the out-of-focus neuropil contamination factor, with an estimated value of ~ 0.7 for this setup. Slow baseline fluctuations were removed by subtracting the eighth percentile value from a 15-s window centered on each frame (Dombeck et al., 2007).

For any given stimulus, a neuron's response was defined by the fluorescence trace in its ROI during the stimulus period:


(1) $$\begin{array}{l} R=\frac{F-F_{0}}{F_{0}} \end{array}$$

where *F* is the instantaneous fluorescence intensity, and *F*_0_ is the mean fluorescence intensity, without visual stimulation.

Two criteria were applied to interpret ROIs as neurons: (1) The size of the ROI was limited to 10–20 μm to match the size of a neuron; (2) The response from the ROI had to pass a signal-to-noise ratio (SNR) of 0.35 (Li and Meister, 2023),


(2) $$\begin{array}{l} SNR=\frac{\text{Var}{\left[{\langle C\rangle }_{r}\right]}_{t}}{{\langle \text{Var}{\left[C\right]}_{r}\rangle }_{t}} \end{array}$$

where *C* is the *N*_*t*_ (time samples) × *N*_*r*_ (stimulus repetitions) response matrix, with *t* = 1, …, *N*_*t*_ and *r* = 1, …, *N*_*r*_; 〈·〉_*r*_ and 〈·〉_*t*_ are the means over repetitions and time respectively, while Var[·]_*r*_ and Var[·]_*t*_ are the corresponding variances. All ROIs meeting these criteria were selected for further analysis, yielding a total of 3,414 neurons, including 490 neurons from four wild-type mice, 337 neurons from one Vglut2-ires-Cre mouse, 1,085 neurons from three Vgat-ires-Cre mice, 720 neurons from three Tac1-IRES2-Cre-D mice, 485 neurons from four Rorb-IRES2-Cre-D mice, and 297 neurons from one Ntsr1–GN209–Cre mouse.

#### 4.5.2 Quantification of functional properties

The functional properties introduced in Figure 4 are defined as follows.

The response to motion (RtM) is defined as the largest absolute response amplitude during moving-bar stimulation. For neurons suppressed by motion, this will be negative.

To quantify the tuning of a neuron to motion directions and orientations, the direction selectivity index (DSI) and orientation selectivity index (OSI) were defined as the normalized amplitude of the response-weighted vector sum of all directions and orientations, respectively:


(3) $$DSI=\frac{\left|\sum_{k}R(\rho_{k})\times {\text{e}}^{i\rho_{k}}\right|}{\sum_{k}R(\rho_{k})}$$

(4) $$OSI=\frac{\left|\sum_{k}R(\theta_{k})\times {\text{e}}^{2i\theta_{k}}\right|}{\sum_{k}R(\theta_{k})}$$

where ρ_*k*_ and θ_*k*_ are the *k*th direction and orientation in radians, and *R*(ρ_*k*_) and *R*(θ_*k*_) are the corresponding peak responses.

To quantify habituation to the expanding black disc, the habituation index (HI) was calculated:


(5) $$\begin{array}{l} HI=\frac{R_{1}-R_{10}}{R_{1}+R_{10}} \end{array}$$

where *R*_1_ and *R*_10_ are the peak response to the first and tenth looming stimulus, respectively.

To quantify the preference for expanding black discs vs. receding white discs, the looming selectivity index (LSI) was calculated:


(6) $$\begin{array}{l} LSI=\frac{R_{\text{k}}-R_{\text{w}}}{R_{\text{k}}+R_{\text{w}}} \end{array}$$

where *R*_k_ is the peak response to the black expanding disc, and *R*_w_ is the peak response to the white receding disc.

To quantify the preference for moving stimuli over flashing stimuli, the motion selectivity index (MSI) was calculated:


(7) $$\begin{array}{l} MSI=\frac{R_{\text{m}}-R_{\text{f}}}{R_{\text{m}}+R_{\text{f}}} \end{array}$$

where *R*_m_ is the peak response to the moving bar at the preferred direction and *R*_f_ is the peak response to the flashing chirp stimulus.

To quantify contrast preference, the contrast selectivity index (CSI) was calculated:


(8) $$\begin{array}{l} CSI=\frac{R_{\text{On}}-R_{\text{Off}}}{R_{\text{On}}+R_{\text{Off}}} \end{array}$$

where *R*_On_ is the peak response to the flashing white square and *R*_Off_ is the peak response to the flashing black square.

To quantify whether neurons show transient or sustained responses to flash stimuli, the peak-final selectivity index (PFSI) was calculated:


(9) $$\begin{array}{l} PFSI=\frac{R_{\text{peak}}-R_{\text{final}}}{R_{\text{peak}}+R_{\text{final}}} \end{array}$$

where *R*_peak_ is the peak response to the flashing white/black square that elicited larger responses, and *R*_final_ is the final response to that stimulus.

To quantify preference for the flashing frequency, the frequency selectivity index (FSI) was calculated:


(10) $$\begin{array}{l} FSI=\frac{R_{\text{low}}-R_{\text{high}}}{R_{\text{low}}+R_{\text{high}}} \end{array}$$

where *R*_low_ is the peak response in the first 3 s to the flashing frequency modulation, and *R*_high_ is the peak response in the last 2 s to the frequency modulation.

The response after frequency modulation (RaFM) was measured as the difference between the response amplitude at 1.6 s after the stop of the frequency modulation and the baseline. Similarly, the response after amplitude modulation (RaAM) was measured as the difference between the response amplitude at 1.6 s after the stop of the amplitude modulation and the baseline.

The best stimulation size (BSS) was defined as the size of the flashing black disc that elicited the largest responses.

To quantify the surround suppression, the surround suppression index (SSI) was calculated:


(11) $$\begin{array}{l} SSI=\frac{R_{\text{small}}-R_{\text{large}}}{R_{\text{small}}+R_{\text{large}}} \end{array}$$

where *R*_small_ is the peak response to the flashing black disc with a diameter of 2 degrees, and *R*_large_ is the peak response to the flashing black disc with a diameter of 32 degrees.

To quantify the color preference, the blue-green index (BGI) was calculated:


(12) $$\begin{array}{l} BGI=\frac{R_{\text{b}}-R_{\text{g}}}{R_{\text{b}}+R_{\text{g}}} \end{array}$$

where *R*_b_ is the response to the flashing blue stimulus, and *R*_g_ is the response to the flashing green stimulus.

To quantify the receptive field size (RFS), the calcium responses at 11 × 11 locations were fitted with a 2-D Gaussian function (Equation 13),


(13) $$f=A\cdot {\text{e}}^{-\frac{{\left(\left(x-E)\cos(D)-(y-F)\sin(D\right)\right)}^{2}}{2B^{2}}-\frac{{\left((x-E)\sin(D)+(y-F)\cos(D)\right)}^{2}}{2C^{2}}}+G$$

The RF size is defined as the area at the half of maximum, which equals π·2ln2·*B*·*C*. The analysis of the RF was performed only if the coefficient of determination for this fit was below 0.5 (Figure 4A). The RF size and position of neurons with a coefficient of determination larger than 0.5 are shown in Figures 4B, C.

#### 4.5.3 Construction of the feature matrix

The dimensionality of the calcium response traces was reduced by approximating them with a weighted sum of features. For each neuron, the responses to all four natural movies was normalized to [0, 1], and then the optimal features were extracted with principal components analysis (PCA) from the response matrix.


(14) $$\begin{array}{l} \text{D}=\text{X}\text{F} \end{array}$$

where **D** (*N*_neuron_ × *N*_time_) is the matrix of neuronal responses to natural movies, **F**(*N*_feature_ × *N*_time_) is the correlation matrix with the time course corresponding to each feature, and **X**(*N*_neuron_ × *N*_feature_) is the feature matrix that carries weight coefficients for each feature.

Sixteen features, which explain 70% of the variance in the data, were extracted from the responses to natural movies. Each feature was normalized so that the mean was 0 and the standard deviation was 1.

#### 4.5.4 Clustering of the feature matrix

Neuronal responses were clustered by applying a Gaussian mixture model (GMM) to the feature matrix **X**.


(15) $$\begin{array}{l} p\left(x\right)=\overset{K}{\underset{i=1}{\sum }}\phi_{i}N\left(x|\mu_{i},\sigma_{i}\right) \end{array}$$

(16) $$\begin{array}{l} N\left(x|\mu_{i},\sigma_{i}\right)=\frac{1}{\sigma_{i}\sqrt{2\pi }}\exp\left(-\frac{{\left(x-\mu_{i}\right)}^{2}}{2\sigma_{i}^{2}}\right) \end{array}$$

(17) $$\begin{array}{l} \overset{K}{\underset{i=1}{\sum }}\phi_{i}=1 \end{array}$$

where *p*(*x*) is the probability density of the feature vector *x*, *K* is the number of component Gaussian functions, and ϕ_*i*_ is the weight for *i*^th^ Gaussian function *N*(*x*|μ_*i*_, σ_*i*_) in the feature space. Parameters were optimized using the EM algorithm (sklearn.mixture.GaussianMixture in the package scikit-learn). The number of components was varied from 2 to 50, and the quality was evaluated with the Bayesian information criterion (BIC) (Kass and Raftery, 1995):


(18) $$\begin{array}{l} BIC=-2\ln L+k\ln n \end{array}$$

where *L* = *p*(*x*|θ, *M*), is the maximized likelihood of model *M*, *x* is the observed data, θ are the parameters that maximize the likelihood, *k* is the number of parameters in the model, and *n* is the number of neurons. The smallest BIC was chosen from the EM fit with 1,000 random initial states, which was plotted against the number of components in Figure 2A.

To evaluate the stability of clusters, 90% of the dataset was sub-sampled 1,000 times and fitted with a GMM using the best cluster number determined from the full dataset (Hennig, 2007). For each original cluster, its Jaccard similarity coefficient (JSC) with the subsets was calculated,


(19) $$\begin{array}{l} JSC=\frac{1}{N}\overset{N}{\underset{i=1}{\sum }}\underset{j}{\max}\left\{\frac{\left|C_{\text{full}}\cup C_{\text{sub}}^{j}\right|}{\left|C_{\text{full}}\cap C_{\text{sub}}^{j}\right|}\right\} \end{array}$$

where *N* is the number of subsets, *C*_full_ is the cluster in the full dataset, and $C_{\text{sub}}^{j}$ is the *j*th cluster in one subset. Clusters with JSC below 0.5 were considered unstable. All clusters were stable (Figure 2E).

To assess the robustness of clustering, the probability that a pair of cells are classified into the same cluster in different subsets was measured, and the co-association matrix (CAM) was calculated (Fred and Jain, 2005):


(20) $$\begin{array}{l} CAM\left(i,j\right)=\frac{n_{i,j}}{N} \end{array}$$

where *n*_*i, j*_ is the number of times that the pair (*i, j*) is assigned to the same cluster in *N* subsets. The between-cluster rate is defined as the cluster-wise average of the co-association matrix.

To plot the dendrogram, a linkage algorithm (scipy.cluster.hierarchy.linkage) to the means of different clusters in the feature space was applied, with the distance between two clusters defined as the Euclidean distance with Ward's minimum variance method.

#### 4.5.5 Relative selectivity index

Relative selectivity index (RSI) is defined as the difference of functional property between one type and a reference number.


(21) $$\begin{array}{l} RSI\left(i,j\right)=F_{i,j}-F_{i,ref} \end{array}$$

where *F*_*i,j*_ is functional property *i* of type *j* and *F*_*i,ref*_ is the reference of functional property *i*. The reference numbers are RtM: 0, DSI: 0.15, OSI: 0.15, HI: 0, LSI: 0, MSI: 0, CSI: 0, PFSI: 0.5, FSI: 0, RaFM: 0, RaAM: 0, BSS: 8, SSI: 0, BGI: 0, RFS: 90 deg^2^.

#### 4.5.6 Analysis of the anatomical arrangement of functional cell types

For the results on anatomical arrangement (Figure 4), only recording sessions with >5 neurons in a field of view were included. The density recovery profile (DRP) plots the probability of finding a cell per unit area as a function of distance from a cell of the same type (Rodieck, 1991). First, the ROI was defined as the convex hull of all neurons in an image, within which, the distances from each reference cell to all of the other cells were histogrammed:


(22) $$\begin{array}{l} N\left(r\right)\text{Δ}r=\text{average number of cells at radii between }r\text{ and }r+\text{Δ}r \end{array}$$

Then the average area *A*(*r*)Δ*r* at the distance between *r* and *r* + Δ*r* from any reference point in the window was measured, and the DRP was calculated as


(23) $$\begin{array}{l} \rho \left(r\right)=\frac{N\left(r\right)}{A\left(r\right)} \end{array}$$

## Data Availability

Code is available in https://github.com/yatangli/Li_NaturalMovies_2025. Data are available in https://zenodo.org/records/14885567.

## References

[B1] Ahmadlou M. Tafreshiha A. Heimel J. A. (2017). Visual cortex limits pop-out in the superior colliculus of awake mice. Cereb. Cortex 27, 5772–5783. 10.1093/cercor/bhx254 29029071 PMC5939206

[B2] Baden T. Berens P. Franke K. Román Rosón M. Bethge M. Euler T.. (2016). The functional diversity of retinal ganglion cells in the mouse. Nature 529, 345–350. 10.1038/nature16468 26735013 PMC4724341

[B3] Barchini J. Shi X. Chen H. Cang J. (2018). Bidirectional encoding of motion contrast in the mouse superior colliculus. eLife 7:e35261. 10.7554/eLife.35261 29963987 PMC6050041

[B4] Bogadhi A. R. Hafed Z. M. (2023). Express detection of visual objects by primate superior colliculus neurons. Sci. Rep. 13:21730. 10.1038/s41598-023-48979-5 38066070 PMC10709564

[B5] Bolton A. D. Murata Y. Kirchner R. Kim S.-Y. Young A. Dang T.. (2015). A Diencephalic dopamine source provides input to the superior colliculus, where D1 and D2 receptors segregate to distinct functional zones. Cell Rep. 13, 1003–1015. 10.1016/j.celrep.2015.09.046 26565913

[B6] Cang J. Savier E. Barchini J. Liu X. (2018). Visual function, organization, and development of the mouse superior colliculus. Ann. Rev. Vis. Sci. 4, 239–262. 10.1146/annurev-vision-091517-034142 29852095

[B7] Deng J. Dong W. Socher R. Li L.-J. Li K. Fei-Fei L.. (2009). “ImageNet: a large-scale hierarchical image database,” in 2009 IEEE Conference on Computer Vision and Pattern Recognition (Miami, FL: IEEE), 248–255. 10.1109/CVPR.2009.5206848

[B8] Dombeck D. A. Khabbaz A. N. Collman F. Adelman T. L. Tank D. W. (2007). Imaging large-scale neural activity with cellular resolution in awake, mobile mice. Neuron 56, 43–57. 10.1016/j.neuron.2007.08.003 17920014 PMC2268027

[B9] Fred A. L. N. Jain A. K. (2005). Combining multiple clusterings using evidence accumulation. IEEE Trans. Pattern Anal. Mach. Intell. 27, 835–850. 10.1109/TPAMI.2005.113 15943417

[B10] Gerfen C. R. Paletzki R. Heintz N. (2013). GENSAT BAC cre-recombinase driver lines to study the functional organization of cerebral cortical and basal ganglia circuits. Neuron 80, 1368–1383. 10.1016/j.neuron.2013.10.016 24360541 PMC3872013

[B11] G'´obel W. Helmchen F. (2007). *In vivo* calcium imaging of neural network function. Physiology 22, 358–365. 10.1152/physiol.00032.2007 18073408

[B12] Harris J. A. Hirokawa K. E. Sorensen S. A. Gu H. Mills M. Ng L. L.. (2014). Anatomical characterization of Cre driver mice for neural circuit mapping and manipulation. Front. Neural Circ. 8:76. 10.3389/fncir.2014.00076 25071457 PMC4091307

[B13] Hennig C. (2007). Cluster-wise assessment of cluster stability. Comput. Stat. Data Anal. 52, 258–271. 10.1016/j.csda.2006.11.025

[B14] Hubel D. H. Wiesel T. N. (1962). Receptive fields, binocular interaction and functional architecture in the cat's visual cortex. J. Physiol. 160, 106–154. 10.1113/jphysiol.1962.sp006837 14449617 PMC1359523

[B15] Kaifosh P. Zaremba J. D. Danielson N. B. Losonczy A. (2014). SIMA: python software for analysis of dynamic fluorescence imaging data. Front. Neuroinform. 8:80. 10.3389/fninf.2014.00080 25295002 PMC4172099

[B16] Kass R. E. Raftery A. E. (1995). Bayes factors. J. Am. Stat. Assoc. 90, 773–795. 10.1080/01621459.1995.10476572

[B17] Kerlin A. M. Andermann M. L. Berezovskii V. K. Reid R. C. (2010). Broadly tuned response properties of diverse inhibitory neuron subtypes in mouse visual cortex. Neuron 67, 858–871. 10.1016/j.neuron.2010.08.002 20826316 PMC3327881

[B18] Knierim J. J. van Essen D. C. (1992). Neuronal responses to static texture patterns in area V1 of the alert macaque monkey. J. Neurophysiol. 67, 961–980. 10.1152/jn.1992.67.4.961 1588394

[B19] Kuffler S. W. (1953). Discharge patterns and functional organization of mammalian retina. *J*. Neurophysiol. 16, 37–68. 10.1152/jn.1953.16.1.37 13035466

[B20] Lee K. H. Tran A. Turan Z. Meister M. (2020). The sifting of visual information in the superior colliculus. eLife 9:e50678. 10.7554/eLife.50678 32286224 PMC7237212

[B21] Li Y.-t. Meister M. (2023). Functional cell types in the mouse superior colliculus. eLife 12:e82367. 10.7554/eLife.82367 37073860 PMC10121220

[B22] Li Y.-t. Turan Z. Meister M. (2020). Functional architecture of motion direction in the mouse superior colliculus. Curr. Biol. 30, 3304–3315.e4. 10.1016/j.cub.2020.06.023 32649907 PMC8221388

[B23] Li Z. Wu R. Li Y.-t. (2023). Calcium imaging in mouse superior colliculus. J. Vis. Exp. 194:e65181. 10.3791/65181 37154575

[B24] Liu Y. Savier E. L. DePiero V. J. Chen C. Schwalbe D. C. Abraham-Fan R.-J.. (2023). Mapping visual functions onto molecular cell types in the mouse superior colliculus. Neuron 111, 1876–1886.e5. 10.1016/j.neuron.2023.03.036 37086721 PMC10330256

[B25] May P. J. (2006). The mammalian superior colliculus: laminar structure and connections. Prog. Brain Res. 151, 321–378. 10.1016/S0079-6123(05)51011-2 16221594

[B26] Mooney R. D. Huang X. Shi M. Y. Bennett-Clarke C. A. Rhoades R. W. (1996). “Chapter 4 Serotonin modulates retinotectal and corticotectal convergence in the superior colliculus,” in Progress in Brain Research, Volume 112 of Extrageniculostriate Mechanisms Underlying Visually-Guided Orientation Behavior, eds. M. Norita, T. Bando, and B. E. Stein (Amsterdam: Elsevier), 57–69. 10.1016/S0079-6123(08)63320-8 8979820

[B27] Nishimoto S. Vu A. T. Naselaris T. Benjamini Y. Yu B. Gallant J. L.. (2011). Reconstructing visual experiences from brain activity evoked by natural movies. Curr. Biol. 21, 1641–1646. 10.1016/j.cub.2011.08.031 21945275 PMC3326357

[B28] Pnevmatikakis E. A. Giovannucci A. (2017). NoRMCorre: an online algorithm for piecewise rigid motion correction of calcium imaging data. J. Neurosci. Methods 291, 83–94. 10.1016/j.jneumeth.2017.07.031 28782629

[B29] Rodieck R. W. (1991). The density recovery profile: a method for the analysis of points in the plane applicable to retinal studies. Vis. Neurosci. 6, 95–111. 10.1017/S095252380001049X 2049333

[B30] Sanes J. R. Masland R. H. (2015). The types of retinal ganglion cells: current status and implications for neuronal classification. Annu. Rev. Neurosci. 38, 221–246. 10.1146/annurev-neuro-071714-034120 25897874

[B31] Savier E. L. Chen H. Cang J. (2019). Effects of locomotion on visual responses in the mouse superior colliculus. J. Neurosci. 39, 9360–9368. 10.1523/JNEUROSCI.1854-19.2019 31570535 PMC6867823

[B32] Wang T. Lee T. S. Yao H. Hong J. Li Y. Jiang H.. (2024). Large-scale calcium imaging reveals a systematic V4 map for encoding natural scenes. Nat. Commun. 15:6401. 10.1038/s41467-024-50821-z 39080309 PMC11289446

[B33] White B. J. Berg D. J. Kan J. Y. Marino R. A. Itti L. Munoz D. P.. (2017). Superior colliculus neurons encode a visual saliency map during free viewing of natural dynamic video. Nat. Commun. 8:14263. 10.1038/ncomms14263 28117340 PMC5286207

[B34] Wu R. Xu J. Li C. Zhang Z. Li L.-y. Li Y.-t. (2024). Preference-independent encoding of visual saliency within but not cross features in the mouse superior colliculus. bioRxiv. 10.1101/2024.03.04.583246

[B35] Yan Y. Zhaoping L. Li W. (2018). Bottom-up saliency and top-down learning in the primary visual cortex of monkeys. *Proc. Natl*. Acad. Sci. 115, 10499–10504. 10.1073/pnas.1803854115 30254154 PMC6187116

[B36] Yu G. Katz L. N. Quaia C. Messinger A. Krauzlis R. J. (2024). Short-latency preference for faces in primate superior colliculus depends on visual cortex. Neuron 112, 2814–2822.e4. 10.1016/j.neuron.2024.06.005 38959893 PMC11343682

[B37] Zhang Y. Kim I.-J. Sanes J. R. Meister M. (2012). The most numerous ganglion cell type of the mouse retina is a selective feature detector. *Proc. Natl*. Acad. Sci. 109, E2391–E2398. 10.1073/pnas.1211547109 22891316 PMC3437843

